# The bioinformatics and experimental analysis of AlkB family for prognosis and immune cell infiltration in hepatocellular carcinoma

**DOI:** 10.7717/peerj.12123

**Published:** 2021-09-01

**Authors:** Bi Peng, Yuanliang Yan, Zhijie Xu

**Affiliations:** 1 Department of Pathology, Xiangya Hospital, Central South University, Changsha, Hunan, China; 2 Department of Pharmacy, Xiangya Hospital, Central South University, Changsha, Hunan, China; 3 National Clinical Research Center for Geriatric Disorders, Xiangya Hospital, Central South University, Changsha, Hunan, China

**Keywords:** Hepatocellular carcinoma, AlkB family, Molecular profiles, Prognosis, Immune cell infiltration

## Abstract

**Background:**

Serving as N6-methyladenosine demethylases, the AlkB family is involved in the tumorigenesis of hepatocellular carcinoma (HCC). However, the molecular profiles and clinical values of the AlkB family in HCC are not well known.

**Methods:**

Several bioinformatics tools and *in vitro* experiments were used to identify the immune-related profiles and prognostic values of AlkB family in HCC.

**Results:**

In this study expression levels of ALKBH1/2/3/4/7 were all remarkably increased in HCC tissues when compared with normal tissues. Quantitative PCR (qPCR) and immunohistochemistry were used to validate the expression of AlkB family members in HCC tissues and normal liver tissues. In addition, high expression levels of ALKBH4 were negatively correlated with overall survival (OS) and disease-free survival (DFS) in patients with HCC. Increased ALKBH4 was also associated with pathological stage in HCC patients. The molecular profiles of AlkB family in HCC were mainly associated with peptidyl-serine modification, peptidyl-tyrosine modification, regulation of metal ion transport, etc. Furthermore, tumor-infiltrating immune cell analysis indicated that ALKBH1/2/3/4/5/6/7/8 and FTO were related to the infiltration of different immune cell, such as CD8+ T cells, macrophages, neutrophils, dendritic cells and CD4+ T cells. We also discovered that the methylation levels of ALKBH1/2/4/5/6/8 and FTO were remarkably reduced in HCC tissues.

**Conclusions:**

Collectively, our findings may deepen the understanding of specific molecular profiles of the AlkB family in HCC pathology. In particular, ALKBH4 could serve as a promising prognostic candidate for treating HCC, and these results might potentiate the development of more reliable therapeutic strategies for patients with HCC.

## Introduction

Hepatocellular carcinoma (HCC), accounting for 75%–85% of liver cancer cases, is one of the leading causes of cancer-associated deaths and it is frequently diagnosed worldwide, with approximately 841,000 new cases and 782,000 deaths yearly. The incidence of HCC has gradually increased in recent years (Feng et al., 2020; Huang et al., 2020). Currently, many scientific researchers have developed many comprehensive clinical therapeutic regimens for HCC, such as liver transplantation (LT), surgical resection, ablation treatment, arterial embolism, and systemic therapies (Akateh et al., 2019). However, to effectively improve the prognosis of patients with HCC, better treatments and novel biomarkers need to be further explored.

The AlkB family of Fe (II)- and *α*-ketoglutarate-dependent dioxygenases are demethylases, playing an important role in removing epigenetic inheritance information. They are closely related to the occurrence and development of many cancers. The AlkB family includes ALKBH1, ALKBH2, ALKBH3, ALKBH4, ALKBH5, ALKBH6, ALKBH7, ALKBH8 and FTO, and they are homologous enzymes (Cai et al., 2021; Wu et al., 2021). The AlkB family mediates many modification processes and stabilizes transcription and translation (Xu et al., 2020a). In addition, they also participate in DNA damage repair (Anindya, 2020), RNA and fatty acid metabolism and histone demethylation (Pilzys et al., 2019). It is worth noting that many studies have shown that the AlkB family is involved in the development of cancers, and the expression profiles of the AlkB family are different in various cancers (Geng et al., 2020). Accumulating evidence suggests that the AlkB family is a promising therapeutic biomarker for various cancers. Therefore, it is important to identify specific molecular profiles of the AlkB family.

Previous results have shown partial functions of the AlkB family in human cancers. However, the specific molecular profiles of AlkB family in HCC biology have not been well clarified, which is a major problem and worth attracting our attention. The AlkB family can be analyzed from multiple levels because of improvements in genetic testing and bioinformatics databases.

## Materials & Methods

### RNA extraction and quantitative PCR (qPCR)

Altogether 13 pairs of formalin-fixed, paraffin-embedded (FFPE) specimens of HCC and adjacent tissues were collected from Department of Pathology, Xiangya Hospital (Changsha, China). The ethics of our study has been approved by the Ethical Committee of Xiangya Hospital of Central South University. The ethical approval number is 202109083. Total RNA was extracted from FFPE tissue specimens using PureLink FFPE RNA Isolation Kit (K156002; Invitrogen, Waltham, MA, USA) according to the manufacturer’s protocol, followed by cDNA synthesis using a PrimeScript RT reagent kit (6210, Takara, China). The quantitative PCR (qPCR) was conducted with iTaq Universal SYBR green Supermix (172-5850; Bio-Rad, Hercules, CA, USA). *β*-actin was used as an internal control. The sequences of all primers used in this report are provided in Table 1. According to the previous reports (Buford et al., 2020; Li et al., 2020b; Pandelides et al., 2020; Zhou et al., 2020), the relative expression levels of AlkB family members were determined using the 2 ^−ΔΔCT^ method.

**Table 1:** The primers sequence of AlkB family members.

Gene	Forward primer	Reverse primer
ALKBH1	5′-AGAAGCGACTAAACGGAGACC-3′	5′-GGGAAAGGTGTGTAATGATCTGC-3′
ALKBH2	5′-GACTGGACAGACCTTCAAC-3′	5′-AGGAGACAGAGGCAATGG-3′
ALKBH3	5′-AGATGTACTGGTTCCCTGGC-3′	5′-CCTCACGGAACACATGGTAG-3′
ALKBH4	5′-GGTCAGCCTCAACCTCCTGT-3′	5′-TATCACGCTGTCCACCAAGG-3′
ALKBH5	5′-GCTTCAGGGTATGGGAGTTG-3′	5′-TTCCAGGATCTGAGTGGATAGA-3′
ALKBH6	5′-TGGACGGATTGGGTGCAAG-3′	5′-TCGAAGCAAATACTCCTCCTCT-3′
ALKBH7	5′-CCATGAGATCCTTCGGGATGA-3′	5′-CGGCAGATCACGGAGATGC-3′
ALKBH8	5′-TTAATGCCACCTAACAAGCCG-3′	5′-ATTGAGGGTAACATAGGCTCTCT-3′
FTO	5′-CCCTGTGAGCAGCAACATAAG-3′	5′-CAACCCGACCCAGTCTAAATC-3′

### The protein levels of AlkB family in clinical samples

Base on the immunohistochemistry technology, the Human Protein Atlas (HPA) has been established to study the protein localization and expression in human cancer and normal tissues (Thul & Lindskog, 2018). Here, the HPA was applied to compare the protein expression of AlkB family members in HCC and normal tissues.

### Functional and pathway enrichment analysis

The Gene Ontology (GO) and Kyoto Encyclopedia of Genes and Genomes pathway (KEGG) enrichment analyses (Ju et al., 2019; Ju et al., 2020b) were analyzed to identify the biological functions and signaling pathways of AlkB associated neighboring genes. The GO and KEGG enrichment analyses were performed by WebGestalt (Liao et al., 2019).

### The profiles of AlkB family analyzed with several databases

In this research, several reliable and multifunctional databases were utilized to analyze all aspects of expression profiles of AlkB family members in HCC (Table S1).

GEPIA2, an updated and enhanced web platform, include 198,619 isoforms and 84 cancer subtypes. GEPIA2 not only contains original functions and extends the analysis of transcript level (Tang et al., 2019). In this research, GEPIA2 was applied for analyzing differential RNA expression between HCC and normal tissues and exploring the correlation between the AlkB family and the stages of pathology. The *p* value was set as 0.05, and Student’s *t*-test was used to evaluate the expression and stage of HCC. Additionally, overall survival (OS) and disease-free survival (DFS) curves were also obtained from GEPIA2.

cBioPortal is an accessible and all-sided website that plays an important role in the visualization and analysis of genomics data in dimensions (Gao et al., 2013). Its data are derived from TCGA and contain more than 200 tumor genomes offering multiple analytical functions. In this study, we analyzed the mutational and coexpressed genes of the AlkB family using the Z score threshold of mRNA and protein ±2.0.

TIMER2.0, a comprehensive web resource, aims to analyze the infiltration of tumor immune cells (Li et al., 2020d). It collected and integrated the data of 10,897 tumors from 32 cancer types. In this research, we conducted a “gene module” to analyze the relationship between the expression levels of the AlkB family in HCC and tumor-infiltrating immune cells.

DiseaseMeth version 2.0 is a convenient and swift website that is mainly used for evaluating the level of aberrant DNA methylation of numerous tumors in 32,701 human samples (Xiong et al., 2017). In our studies, the “Analysis module” was utilized to evaluate the level of DNA methylation of the AlkB family in HCC patients.

### Statistical analyses

The statistical tests were analyzed by using SPSS 12.0 software (IBM Analytics). GraphPad Prism 8 (GraphPad Software) was used to draw the bar graphs. The paired-samples *t*-test was used to analyze the data of qPCR. a *p*-value of less than 0.05 was considered to be statistically significant.

## Results

### Abnormal expression of AlkB family in HCC

**Figure 1:**
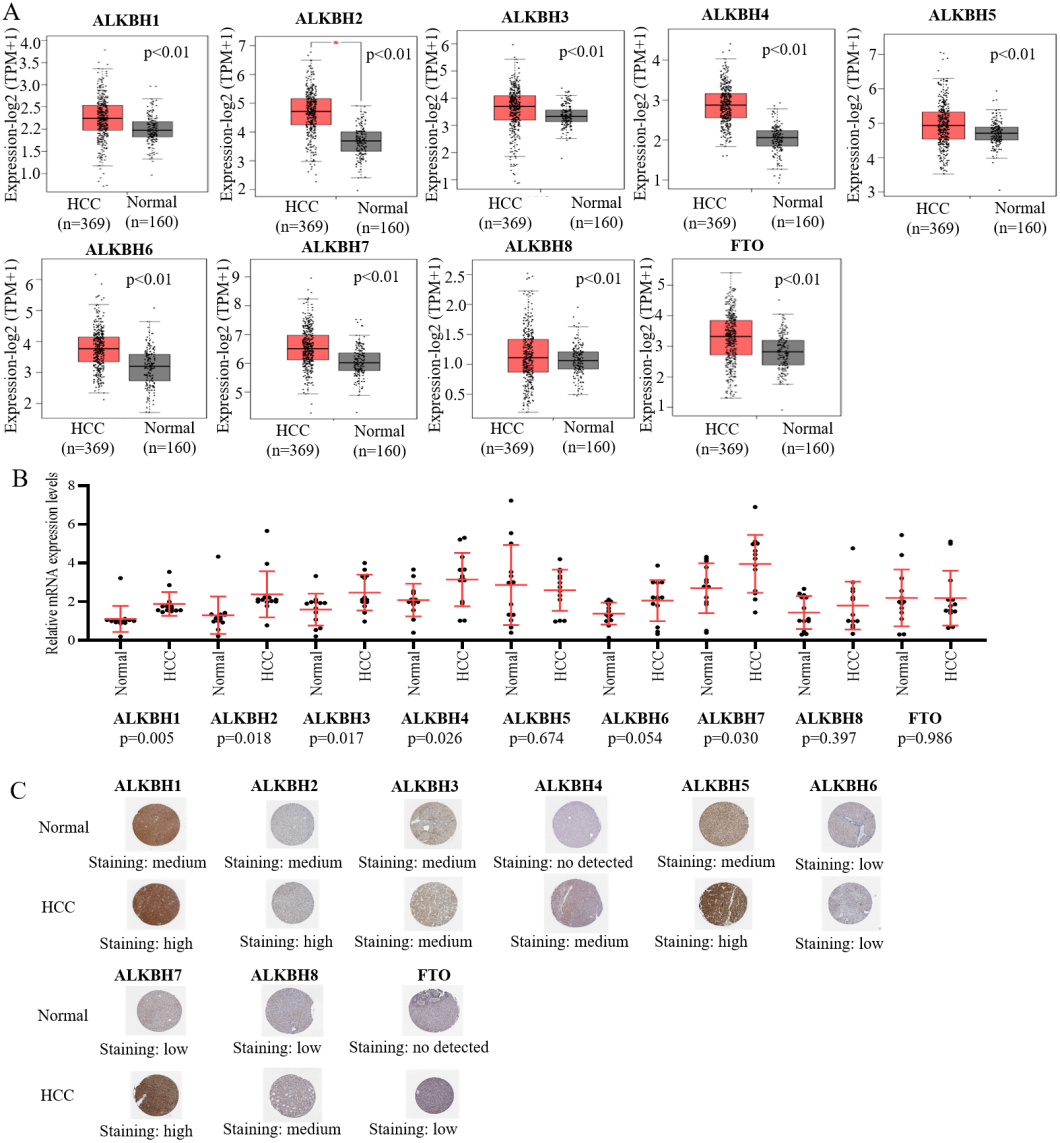
The expression level of AlkB family in HCC tissues. (A) The mRNA expression profiles of AlkB family members in HCC patients from GEPIA2 databases. (B) The mRNA expression level of AlkB family members in the clinical samples. (C) The HPA showed the immunohistochemical analysis of ALKB family in HCC tissue and normal tissue.

**Figure 2:**
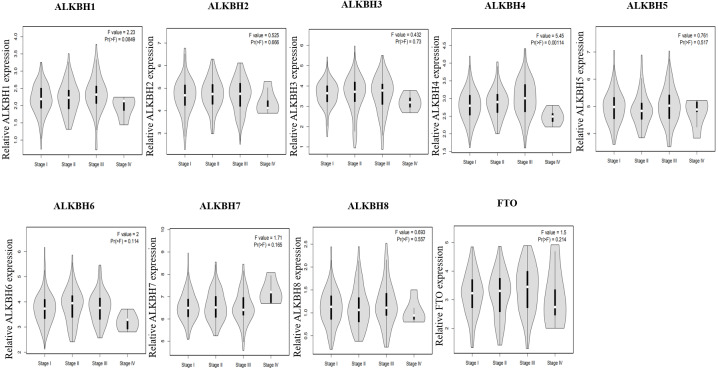
The relationship of AlkB family and pathology stages in HCC patients. The GEPIA2 webtool was used to analyze the relevant between expression levels of AlkB family members and patients’ stages.

We utilized the GEPIA databases to retrieve the AlkB family, and the “Expression DIY module” was used to evaluate the expression level of the AlkB family between HCC and normal hepatic tissues. Fig. 1A shows that the transcriptional levels of ALKBH1/2/3/4/5/6/7/8 (*p* < 0.01) and FTO (*p* < 0.01) were all remarkably increased in HCC tissues when compared with normal tissues. We also evaluated the transcriptional levels of the Alkb family members in the HCC and paired adjacent normal tissues were. The result of qPCR showed that the mRNA expression levels of ALKBH1/2/3/4/7 are higher in tumor samples and lower in normal tissue samples and the others had no statistic difference (Fig. 1B). In addition, we used the HPA database to analyze the protein expression levels of Alkb family in HCC tissues and normal liver tissues. The results showed that ALKBH1/2/4/5/7/8 were medium or high expression in HCC tissues (Fig. 1C), which is consistent with their changes on the transcriptional levels. These findings are similar with other reports. For example, Ma et al. found that the expression level of ALKBH1 in HCC was strikingly elevated (Ma et al., 2019). The relationship between the expression level of the AlkB family and the stages of pathology was then evaluated. With the progression of tumors, ALKBH4 (*p* = 0.00114) was increasingly expressed in the first three stages (Fig. 2). Then, Xiantao Xueshu (https://www.xiantao.love/products) was used to further evaluate the relationship between ALKBH4 expression and HCC. The results revealed that ALKBH4 expression significantly increased with the increase in T stage (odds ratio = 1.629, *p* = 0.02). Increased ALKBH4 expression had the significance on pathological stage despite there was no statistical difference. The correlation between ALKBH4 and other characteristics in HCC patients, including N stage, M stage and histologic grade, needs to be further explored (Table 2). All in all, our results cast new light on the specific molecular profiles of the AlkB family in HCC.

**Table 2:** Demographic characteristics of ALKBH4 expression in HCC.

Characteristics	Total(N)	Odds Ratio (OR)	*P* value
T stage (T2&T3&T4 vs. T1)	371	1.629 (1.082–2.460)	0.020
N stage (N1 vs. N0)	258	2.773 (0.350–56.463)	0.380
M stage (M1 vs. M0)	272	0.319 (0.016–2.525)	0.325
Pathologic stage (Stage II&Stage III&Stage IV vs. Stage I)	350	1.513 (0.994–2.310)	0.054
Histologic grade (G2&G3&G4 vs. G1)	369	1.578 (0.886–2.859)	0.125
Tumor status (With tumor vs. Tumor free)	355	1.456 (0.955–2.224)	0.081
Age (>60 vs. <=60)	373	1.125 (0.749–1.691)	0.570
Gender (Male vs. Female)	374	1.187 (0.769–1.834)	0.439
Vascular invasion (No vs. Yes)	318	0.960 (0.604–1.526)	0.863
Residual tumor (R1&R2 vs. R0)	345	2.087 (0.791–6.119)	0.151
AFP (ng/ml) (>400 vs. <=400)	280	1.548 (0.887–2.726)	0.126
Albumin(g/dl) (>=3.5 vs. <3.5)	300	0.920 (0.536–1.578)	0.760

### The prognostic value of AlkB family in HCC patients

**Figure 3:**
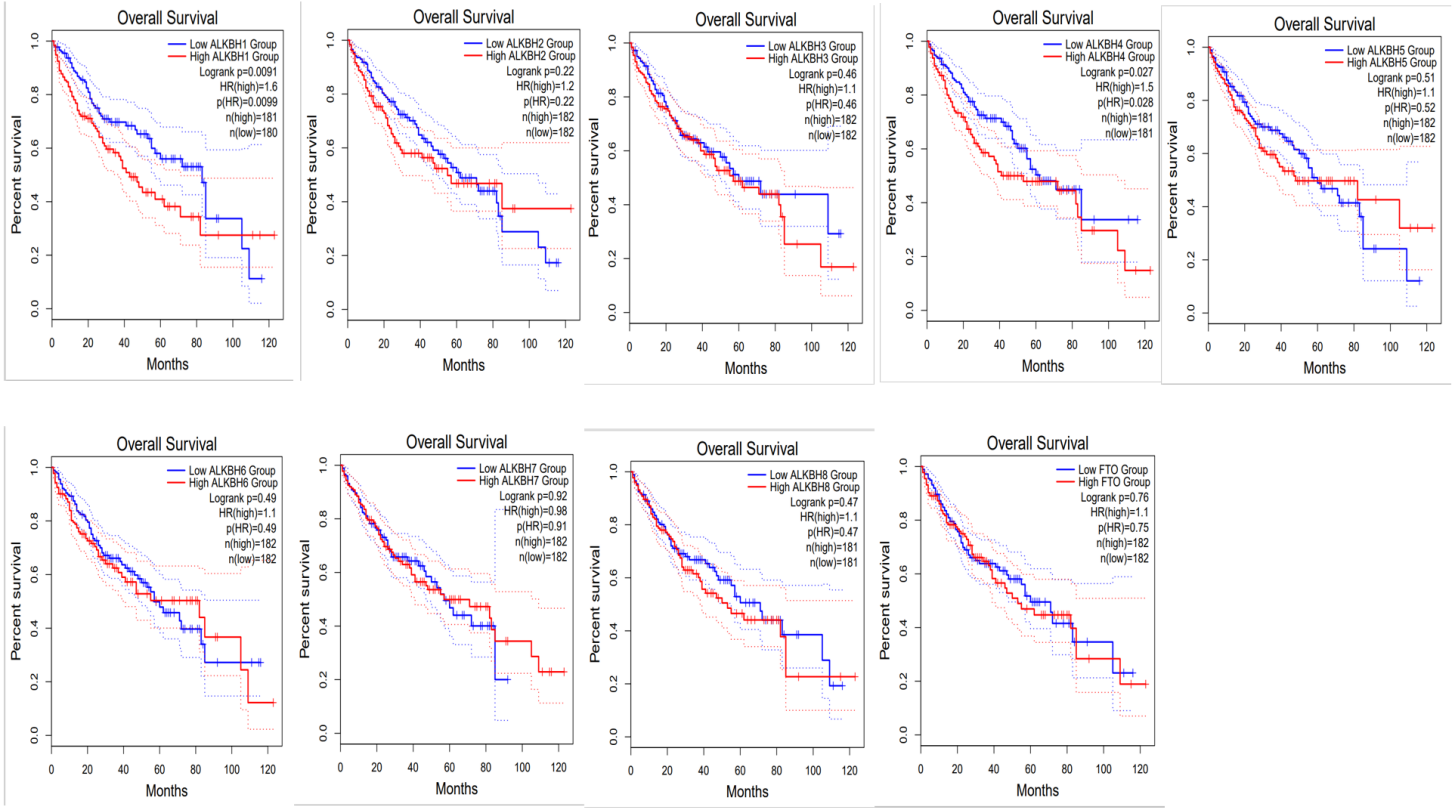
The effect of AlkB family on overall survival of HCC patients. GEPIA2 was used to evaluate the relationship of the AlkB family and OS in HCC patients.

**Figure 4:**
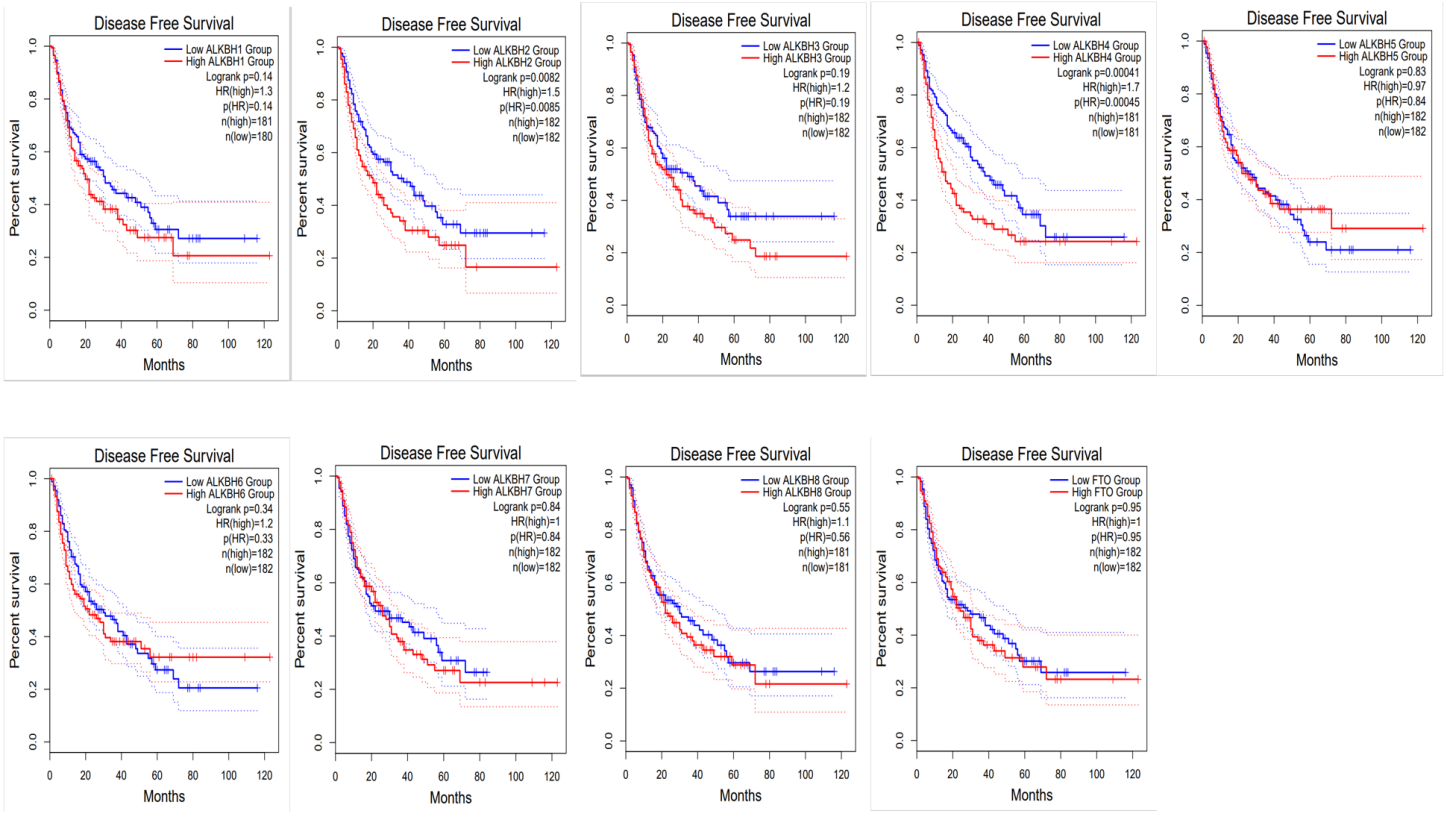
The effect of AlkB family on disease free survival of HCC patients. The relationship of the expression level of the AlkB family and DFS in HCC patients was analyzed by GEPIA2

To identify the prognostic value of different expression levels of the AlkB family, we performed survival analysis and explored the interactive correlations. Figure 3 presents the OS curves. In this study, we found that remarkably increased ALKBH1 (*p* = 0.0099) and ALKBH4 (*p* = 0.028) expression were correlated with a bad prognosis (Fig. 3). Then, we also evaluated the DFS of patients with HCC. We observed that similarly, high expression levels of ALKBH2 (*p* = 0.0085) and ALKBH4 (*p* = 0.00041) were correlated with a poor DFS (Fig. 4). These data indicated the important roles of high expressed ALKBH4 on HCC prognosis.

### Genetic alteration and enriched functional analysis of AlkB family in HCC patients

**Figure 5:**
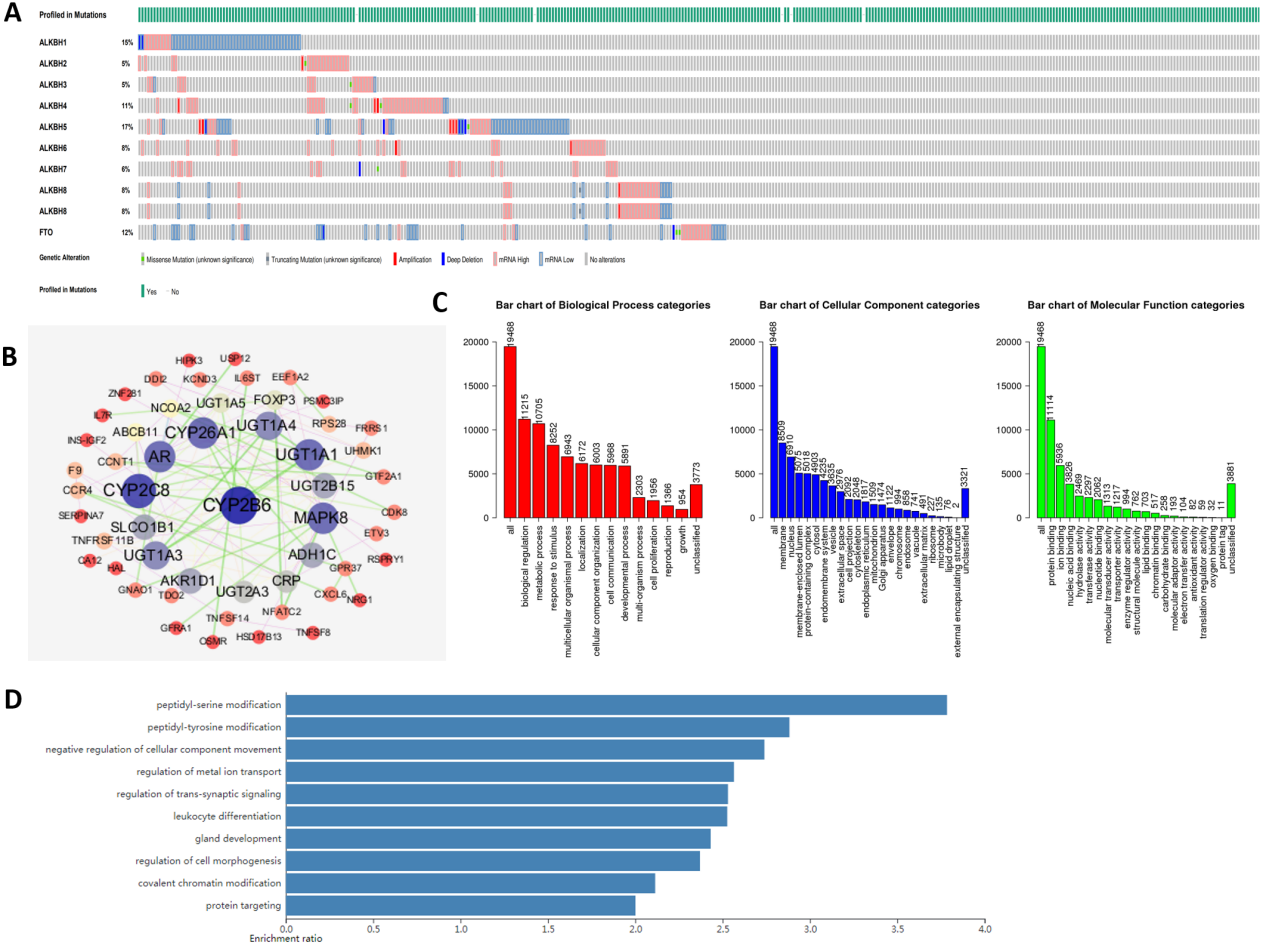
Genetic alterations and interaction analyses of the AlkB family in HCC patients. (A) General mutations of the AlkB family in HCC. (B) The cBioPortal was used to identify the neighboring genes of AlkB family in HCC. (C) Bar chart of the biological process categories, cellular component categories and molecular function categories. (D) Bar chart of the KEGG enrichment results.

At the molecular level, an analysis of genetic alterations was conducted on the AlkB family in HCC based on the cBioPortal database. Our findings hinted that the genetic mutations occurred in ALKBH1/2/3/4/5/6/7/8 and FTO in HCC patients, with altered rates of 15, 5, 5, 11, 17, 8, 6, 8, and 12%, respectively (Fig. 5A). Moreover, we downloaded 19,909 genes related to HCC from the cBioPortal, and 155 genes were screened with *p* < 0.01 and —log ratio—> 0.85 (Table S2). Ultimately, using the protein-protein interaction analysis, we identified approximately 145 neighboring genes of the AlkB family. The hub genes mainly included Cytochrome P450 family 2 subfamily B member 6 (CYP2B6), CYP2C8, CYP26A1 and so on (Fig. 5B).

Then, we conducted gene ontology (GO) enrichment analysis of the interacting gene network of the AlkB family with WebGestalt. Figure 5C shows that in biological process (BP), biological regulation, metabolic process, response to stimulus, multicellular organismal process and localization were the top five most highly enriched categories. Meanwhile, at the cellular level, there were many enriched items, and the top 5 covered the membrane, nucleus, membrane-enclosed lumen, protein-containing complex and cytosol. Moreover, protein binding and ion binding were the richest categories in the molecular function (MF). In addition, Kyoto Encyclopedia of Genes and Genomes (KEGG) enrichment analysis suggested that peptidyl-serine modification, peptidyl-tyrosine modification, negative regulation of cellular component movement, regulation of metal ion transport and regulation of transsynaptic signaling were the main signaling pathways in connection with the abnormal AlkB family members, which might play the potential roles in the occurrence and development of HCC (Fig. 5D).

### Immune cell infiltration under the influence of AlkB family in HCC

**Figure 6:**
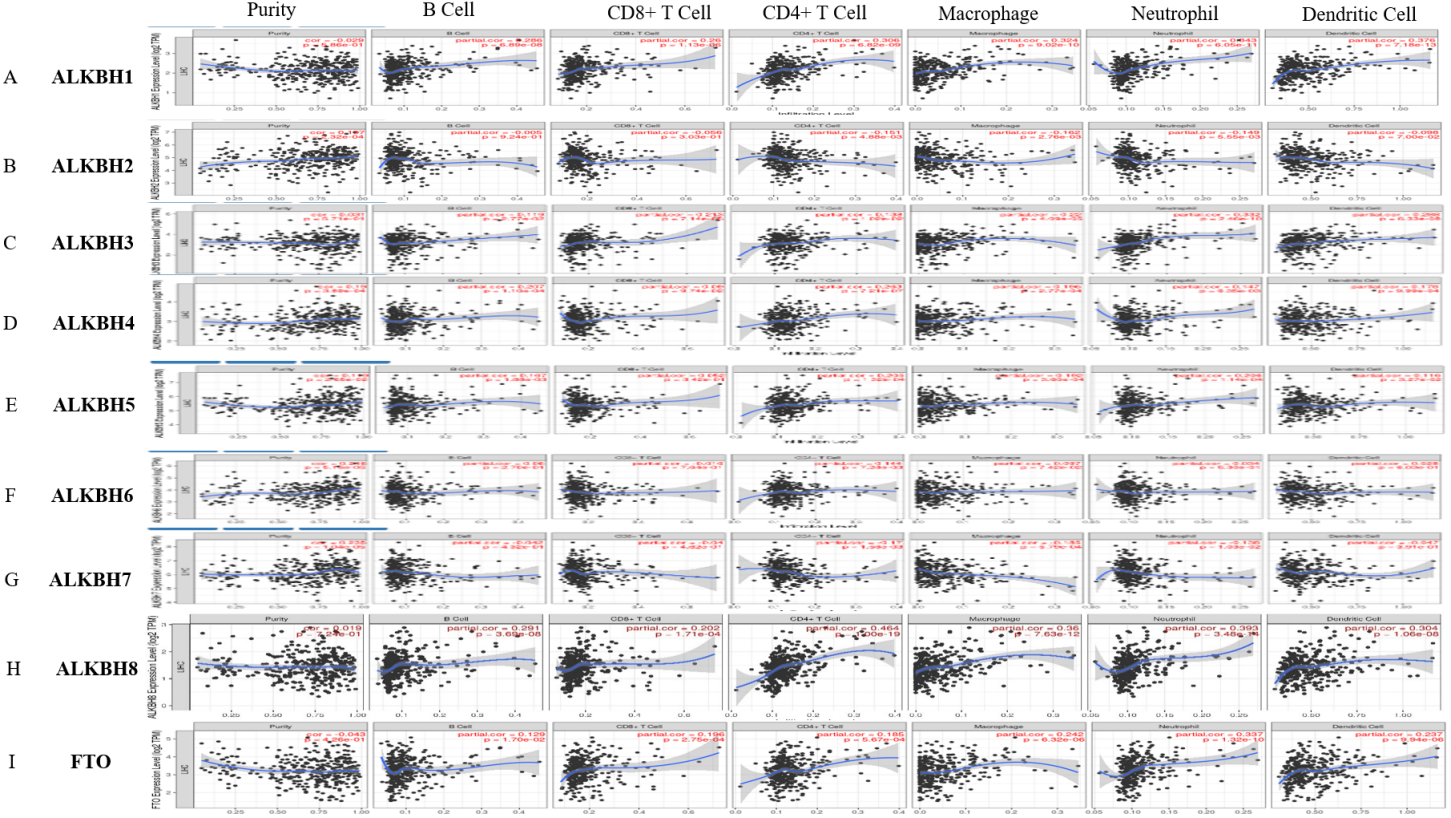
The relationship of AlkB family and tumor-infiltrating immune cells. (A-I) The effect of (A) ALKBH1, (B) ALKBH2, (C) ALKBH3, (D) ALKBH4, (E) ALKBH5, (F) ALKBH6, (G) ALKBH7, (H) ALKBH8 and (I) FTO on the abundance of immune cells in HCC patients.

A reliable correlation analysis between tumor-infiltrating immune cells and the AlkB family was performed based on TIMER (Fig. 6). In detail, ALKBH1 was positively correlated with the infiltration of B cells (Cor =0.286, *p* = 6.89e−08), CD8+ cells (Cor = 0.26, *p* = 1.13e−06), CD4+ cells (Cor = 0.306, *p* = 6.82e−09), macrophages (Cor = 0.324, *p* = 9.02e−10), neutrophil (Cor = 0.843, *p* = 6.05e−11) and dendritic cells (Cor =0.376, *p* = 7.18e−13) (Fig. 6A). ALKBH2 had negative correlations with the infiltration of CD4+ cells (Cor = −0.151, *p* = 4.88e−03) and macrophages (Cor = −0.162, *p* = 2.76e−03) (Fig. 6B). ALKBH3 was positively correlated with the infiltration of CD8+ cells (Cor = 0.213, *p* = 7.14e−05), macrophages (Cor =0.22, *p* = 4.09e−05), neutrophils (Cor = 0.332, *p* = 2.46e−10) and dendritic cells (Cor = 0.288, *p* = 6.33e−08) (Fig. 6C). ALKBH4 had a positive relationship with infiltration of B cells (Cor = 0.207, *p* = 1.10e−04), CD4+ cells (Cor = 0.263, *p* = 7.21e−07), macrophages (Cor = 0.196, *p* = 2.77e−04), neutrophil (Cor = 0.147, *p* = 6.53e−03) and dendritic cells (Cor = 0.178, *p* = 9.99e−04) (Fig. 6D). ALKBH5 was positively associated with the infiltration of B cells (Cor =0.167, *p* = 1.88e−03), CD4+ cells (Cor = 0.205, *p* = 1.32e−04), macrophages (Cor = 0.192, *p* = 3.60e−04), neutrophil (Cor = 0.206, *p* = 1.14e−04) and dendritic cells (Cor = 0.116, *p* = 3.27e−02) (Fig. 6E). Figure 6F indicated positive correlations between ALKBH6 and the infiltration of CD4+ cells (Cor = 0.144, *p* = 7.28e−03). Conversely, negative correlations between ALKBH7 and CD4+ cells (Cor = −0.17, *p* = 1.56e−03), macrophages (Cor = −0.185, *p* = 5.79e−04) and neutrophil (Cor = −0.138, *p* = 1.03e−02) were showed in Fig. 6G. Surprisingly, ALKBH8 and FTO were positively correlated with all six types of immune cells analyzed (Figs. 6H–6I). We further utilized the Cox proportional hazard model to correct for confounders, including B cells, CD8+ T cells, CD4+ T cells, macrophages, neutrophils and the AlkB family. The results indicated that B cells, CD8+ T cells, macrophages and ALKBH4 were related to the prognosis of patients with HCC (Table S3).

### The different methylation levels of AlkB family in HCC

There is a negative correlation between DNA methylation and gene expression levels (Gensous et al., 2020; Kronfol et al., 2020; Wang et al., 2020b; Zhang et al., 2020). In our results, the DNA methylation of ALKBH1/2/4/5 and FTO was decreased in HCC patients compared with healthy persons (*p* < 0.05) (Fig. S1). Therefore, we concluded that higher expression levels of ALKBH1/2/4/5 and FTO in HCC tissues might be due to downregulated DNA methylation levels.

## Discussion

N6-methyladenosine is extensively involved in RNA modification (Zheng, Zhang & Sui, 2020), and it is closely related to the occurrence and development of various cancers (Li et al., 2020a; Xu et al., 2020b). Surprisingly, serving as demethylases, ALKBH5 and FTO facilitate the demethylation process of m6A modification (Toh et al., 2020). In addition, the AlkB family is also involved in DNA repair, transcription and translation, according to previous studies (Baldwin, Admiraal & O’Brien, 2020; Xu et al., 2020a). There are many findings indicated that m6A modification mediated by ALKBH5 and FTO participates in the development of HCC and plays a crucial role in suppressing proliferative and invasive abilities of HCC cells (Bian et al., 2021; Chen et al., 2020). The expression of ALKBH5 and FTO has been proved to be up-regulated in tumor tissues, which implies a poor prognosis of HCC patients (Li et al., 2019; Qu et al., 2021). Apart from that, m6A is evidently connected with HCC immune microenvironment. For example, FTO-mediated m6A demethylation gets involved in the response to anti-programmed cell death-1 (anti-PD-1)-based immunotherapy (Yang et al., 2019). Methyltransferase-like 3 (METTL3) and methyltransferase-like 114 (METTL114), the major m6A RNA methyltransferases, enhance the sensitivity of anti-PD-1 treatment (Wang et al., 2020a). Consequently, the AlkB family participates in the growth, prognosis and immune regulation of cancers from many aspects. However, the specific molecular profiles and detailed roles of the AlkB family in HCC are not well known.

First, we utilized GEPIA2 to explore the expression levels of the AlkB family, and we found that the AlkB family members were all expressed at a higher level compared with normal tissues. We evaluated expression levels in different pathological stages and found that ALKBH4 was increasingly expressed in the first three stages. This hinted that we might estimate the pathological stage of HCC by testing the expression level of ALKBH4. In addition, we observed that high expression of ALKBH4 was correlated with a poor prognosis in HCC. These results provide a novel insight into the vital roles of high expressed ALKBH4 in HCC.

Second, we genetically explored the correlation between the AlkB family and HCC. We found that the whole AlkB family had many genetic alterations in HCC patients, supporting again that the AlkB family might be involved in the tumorigenesis and progression of HCC.

Third, we performed GO enrichment analysis based on the WebGestalt database. We found that the functions of the AlkB family were mainly associated with peptidyl serine modification, peptidyl-tyrosine modification, negative regulation of cellular component movement, regulation of metal ion transport, regulation of transsynaptic signaling and leukocyte differentiation. As reported by previous studies, metal ion transport, transsynaptic signaling and leukocyte differentiation are related to hepatic tumor growth and progression (Hou et al., 2020; Ren et al., 2018). These results suggested that the AlkB family might participate in the progression of HCC by regulating these signaling pathways.

Then, previous studies showed that immune cell infiltration plays a crucial role in tumorigenesis, prognosis and therapies (Mukherjee et al., 2020; Xu et al., 2020c; Yan et al., 2020). Most T cells and macrophage-mediated phagocytosis are capable of killing tumor cells and preventing tumor growth (Feng et al., 2019). The results of our study showed that the expression levels of ALKBH1, ALKBH3, ALKBH5 and ALKBH8 were positively associated with macrophages, neutrophils, dendritic cells and CD4+ T cells, which suggested partial specific biological profiles of the AlkB family. Growing evidence has indicated that many cancer-associated genes serve as potential immune-associated prognostic biomarkers. The research results of Li et al. (2020c) demonstrated that ALKBH5 plays an important part in recruiting decreasingly of MDSCs and Tregs to restrict tumor growth. Ju et al. (2020a) have illustrated that erythroid 2 like 2 (NFE2L2) expression is positively associated with immune infiltration, which hints a more favorable prognosis in brain lower grade glioma.

Although we have made a comprehensive analysis of AlkB family from many aspects, there are still some issues that need to be addressed. First of all, we collected a small set of samples for the qPCR experiment. Secondly, our results exhibited a remarkable correlation between the expression of ALKBH4 and HCC. However, the specific mechanisms of their interaction remain to be explored. Finally, more *in vitro* and *in vivo* experiments should be conducted to confirm our results.

## Conclusion

In conclusion, this article comprehensively explored the molecular profiles of AlkB family through multiple levels of analysis based on many bioinformatics databases. Furthermore, the ALKBH4 might show the tumor-promoting effect and negatively affect the prognosis of patients with HCC. These findings can deepen clinicians’ understanding of HCC and could potentiate the development of more accurate and reliable treatments for patients with HCC.

##  Supplemental Information

10.7717/peerj.12123/supp-1 Supplemental Information 1 Several reliable and multifunctional databases were used to comprehensively analyze the AlkB family in HCC

10.7717/peerj.12123/supp-2 Supplemental Information 2 Screened co-expressed genes of the AlkB family in HCC

10.7717/peerj.12123/supp-3 Supplemental Information 3 The cox proportional hazard model of the AlkB family and six immune cells involved in tumor infiltration in HCC

10.7717/peerj.12123/supp-4 Supplemental Information 4 The DNA methylation levels of AlkB family in HCC patients

10.7717/peerj.12123/supp-5 Supplemental Information 5 The Raw Data for mRNA expression values

## Additional Information and Declarations

### Data Availability

The following information was supplied regarding data availability: The raw measurements for the qPCR are available in the Supplemental Files.
