# Supplementary material for: The bioinformatics and experimental analysis of AlkB family for prognosis and immune cell infiltration in hepatocellular carcinoma
Source: PeerJ. 2021 Sep 1;9:e12123. doi: 10.7717/peerj.12123 (PMC8418211; doi:10.7717/peerj.12123)
Supplement: Supplemental Information 1 [file peerj-09-12123-s001.docx]

**Table S1.** Several reliable and multifunctional databases were used to comprehensively analyze the AlkB family in HCC

| Databases | Samples | URL | Refs |
| --- | --- | --- | --- |
| The Human Protein Atlas | Tissues | https://www.proteinatlas.org/ | (Thul & Lindskog 2018) |
| GEPIA2 | Tissues | http://gepia.cancer-pku.cn/ | (Tang et l., 2019) |
| cBioPortal | Tissues | http://www.cbioportal.org/ | (Gao et al. 2013) |
| TIMER2.0 | - | http://timer.cistrome.org/ | (Li et al. 2020d) |
| DiseaseMeth version 2.0 | - | http://bioinfo.hrbmu.edu.cn/diseasemeth/ | (Xiong et al. 2017) |
| WebGestalt | - | http://www.webgestalt.org/option.php | (Liao et al. 2019) |
