# Supplementary material for: The bioinformatics and experimental analysis of AlkB family for prognosis and immune cell infiltration in hepatocellular carcinoma
Source: PeerJ. 2021 Sep 1;9:e12123. doi: 10.7717/peerj.12123 (PMC8418211; doi:10.7717/peerj.12123)
Supplement: Supplemental Information 2 [file peerj-09-12123-s002.docx]

**Table S2**. Screened co-expressed genes of the AlkB family in HCC.

| Gene | Log Ratio | p-Value | Higher expression in |
| --- | --- | --- | --- |
| DHX33 | -1.06 | 4.73E-13 | Unaltered group |
| AKAP2 | -1.76 | 1.32E-12 | Unaltered group |
| NBPF10 | -1.36 | 5.55E-12 | Unaltered group |
| RALGAPA2 | -1.16 | 2.24E-11 | Unaltered group |
| HIPK3 | -1.29 | 6.05E-11 | Unaltered group |
| GTF2I | -0.86 | 6.21E-11 | Unaltered group |
| TGFBRAP1 | -1.11 | 9.21E-11 | Unaltered group |
| FNIP2 | -1.2 | 9.52E-11 | Unaltered group |
| USP12 | -1.09 | 9.82E-11 | Unaltered group |
| SBNO1 | -1.19 | 1.20E-10 | Unaltered group |
| NCOA2 | -1.32 | 1.55E-10 | Unaltered group |
| CDK8 | -0.9 | 1.98E-10 | Unaltered group |
| LOC284441 | -0.9 | 1.99E-10 | Unaltered group |
| CARNMT1 | -0.93 | 2.23E-10 | Unaltered group |
| SPATA13 | -1.18 | 2.49E-10 | Unaltered group |
| SYNC | 1.8 | 2.54E-10 | Altered group |
| KBTBD7 | -0.87 | 3.38E-10 | Unaltered group |
| RSPRY1 | -0.87 | 3.53E-10 | Unaltered group |
| GOLIM4 | -1.1 | 3.71E-10 | Unaltered group |
| NBPF9 | -1.18 | 4.21E-10 | Unaltered group |
| UGCG | -1.08 | 4.56E-10 | Unaltered group |
| IL6ST | -1.45 | 4.64E-10 | Unaltered group |
| FAM83G | -1.01 | 4.94E-10 | Unaltered group |
| GTF2A1 | -1.04 | 5.11E-10 | Unaltered group |
| PPTC7 | -1.1 | 5.25E-10 | Unaltered group |
| MAPK8 | -0.89 | 6.06E-10 | Unaltered group |
| GLUD2 | -0.91 | 9.11E-10 | Unaltered group |
| CCNT1 | -1.11 | 1.18E-09 | Unaltered group |
| LEPROT | -1.02 | 1.23E-09 | Unaltered group |
| RAPGEF6 | -1.05 | 1.40E-09 | Unaltered group |
| ZNF281 | -0.96 | 1.86E-09 | Unaltered group |
| C9ORF129 | -1.01 | 2.39E-09 | Unaltered group |
| TNFSF8 | -0.92 | 2.49E-09 | Unaltered group |
| LMTK2 | -0.93 | 2.51E-09 | Unaltered group |
| ETV3 | -0.98 | 3.01E-09 | Unaltered group |
| TXLNG | -0.89 | 3.19E-09 | Unaltered group |
| MAFG-DT | 0.91 | 3.20E-09 | Altered group |
| CLDN22 | 1.02 | 3.94E-09 | Altered group |
| NFIC | -0.87 | 7.35E-09 | Unaltered group |
| REST | -1.05 | 8.27E-09 | Unaltered group |
| NIPAL1 | -1.22 | 1.03E-08 | Unaltered group |
| ZKSCAN8 | -1.15 | 1.03E-08 | Unaltered group |
| HACD2 | -1.17 | 1.17E-08 | Unaltered group |
| DDI2 | -1.22 | 1.21E-08 | Unaltered group |
| N4BP2 | -1.03 | 1.46E-08 | Unaltered group |
| PSMC3IP | 0.88 | 1.62E-08 | Altered group |
| EMILIN3 | 1.08 | 1.68E-08 | Altered group |
| HS3ST3B1 | -1.09 | 2.09E-08 | Unaltered group |
| KLHL23 | -1.05 | 2.21E-08 | Unaltered group |
| PROX1 | -1.04 | 3.86E-08 | Unaltered group |
| CCR4 | -0.88 | 6.00E-08 | Unaltered group |
| EFNA2 | -0.86 | 6.04E-08 | Unaltered group |
| RASSF3 | -1.09 | 9.90E-08 | Unaltered group |
| MPZL3 | -0.85 | 1.27E-07 | Unaltered group |
| ANKRD36BP1 | -1 | 1.37E-07 | Unaltered group |
| COL27A1 | -0.92 | 1.57E-07 | Unaltered group |
| SERINC5 | -0.9 | 1.60E-07 | Unaltered group |
| ZDHHC20 | -0.99 | 1.84E-07 | Unaltered group |
| ERN1 | -0.88 | 2.00E-07 | Unaltered group |
| NFATC2 | -1.17 | 2.10E-07 | Unaltered group |
| IPMK | -0.87 | 2.23E-07 | Unaltered group |
| FAT4 | -0.94 | 2.63E-07 | Unaltered group |
| PCDHGA12 | -1.07 | 2.76E-07 | Unaltered group |
| TMEM273 | -0.9 | 4.29E-07 | Unaltered group |
| PCDHGB7 | -0.98 | 4.34E-07 | Unaltered group |
| UHMK1 | -0.96 | 5.44E-07 | Unaltered group |
| BEND3P3 | -0.85 | 6.27E-07 | Unaltered group |
| FRRS1 | -0.92 | 6.78E-07 | Unaltered group |
| NLRP6 | -1.19 | 6.83E-07 | Unaltered group |
| PCDHGB2 | -1.12 | 9.24E-07 | Unaltered group |
| BHLHA15 | -0.88 | 9.64E-07 | Unaltered group |
| ITGB3 | -0.91 | 1.00E-06 | Unaltered group |
| BIRC5 | 0.88 | 1.35E-06 | Altered group |
| WASHC2A | -1.1 | 1.57E-06 | Unaltered group |
| TMEM154 | -0.98 | 1.61E-06 | Unaltered group |
| IL7R | -1.14 | 2.04E-06 | Unaltered group |
| MGAT5 | -0.93 | 2.41E-06 | Unaltered group |
| KIRREL1 | -1.06 | 4.32E-06 | Unaltered group |
| TNFRSF11B | -1.13 | 4.50E-06 | Unaltered group |
| DBH | -0.93 | 4.52E-06 | Unaltered group |
| UGT2A3 | -1.04 | 6.08E-06 | Unaltered group |
| EDIL3 | -1.05 | 6.55E-06 | Unaltered group |
| TNFSF14 | -1.01 | 8.38E-06 | Unaltered group |
| AVPR1A | -1.37 | 8.50E-06 | Unaltered group |
| PLXNC1 | -1.11 | 1.17E-05 | Unaltered group |
| SLCO1B1 | -1.01 | 1.22E-05 | Unaltered group |
| PLGLA | -0.91 | 1.45E-05 | Unaltered group |
| OSMR | -0.85 | 1.53E-05 | Unaltered group |
| TTPA | -0.92 | 3.94E-05 | Unaltered group |
| PCDHGA2 | -1.02 | 4.14E-05 | Unaltered group |
| FAM66D | 0.86 | 4.85E-05 | Altered group |
| RPS28 | 1.21 | 5.07E-05 | Altered group |
| ASIC1 | 0.94 | 5.29E-05 | Altered group |
| FOXP3 | -0.85 | 5.52E-05 | Unaltered group |
| GNAO1 | -1.04 | 6.90E-05 | Unaltered group |
| KCND3 | -1 | 6.92E-05 | Unaltered group |
| COL4A3 | -0.88 | 7.17E-05 | Unaltered group |
| PAPPA2 | -1.15 | 8.83E-05 | Unaltered group |
| HSD17B13 | -1.66 | 9.12E-05 | Unaltered group |
| UGT1A1 | -1.14 | 9.24E-05 | Unaltered group |
| AR | -1.04 | 1.01E-04 | Unaltered group |
| SLIT2 | -0.92 | 1.20E-04 | Unaltered group |
| PCDHGA4 | -0.85 | 1.30E-04 | Unaltered group |
| GBA3 | -1.11 | 1.85E-04 | Unaltered group |
| SLC3A1 | -1.22 | 1.99E-04 | Unaltered group |
| SNORD116-4 | -0.9 | 2.38E-04 | Unaltered group |
| FAM99B | -0.86 | 2.43E-04 | Unaltered group |
| INS-IGF2 | -1.39 | 2.60E-04 | Unaltered group |
| MPPED1 | -0.99 | 2.84E-04 | Unaltered group |
| MAB21L2 | -0.89 | 3.09E-04 | Unaltered group |
| ADRA1A | -0.94 | 3.72E-04 | Unaltered group |
| TDO2 | -1 | 4.65E-04 | Unaltered group |
| HOXC9 | 0.86 | 5.18E-04 | Altered group |
| BICC1 | -1.05 | 5.44E-04 | Unaltered group |
| CYP4F22 | -1.12 | 6.20E-04 | Unaltered group |
| ADGRD1 | -0.96 | 6.50E-04 | Unaltered group |
| HPGD | -1.25 | 7.69E-04 | Unaltered group |
| SORCS2 | -0.9 | 7.94E-04 | Unaltered group |
| IGSF9 | -1 | 8.92E-04 | Unaltered group |
| RASEF | -0.95 | 9.37E-04 | Unaltered group |
| GPR37 | -0.87 | 1.10E-03 | Unaltered group |
| CYP26A1 | -0.85 | 1.15E-03 | Unaltered group |
| F9 | -1.07 | 1.18E-03 | Unaltered group |
| PIK3C2G | -0.91 | 1.22E-03 | Unaltered group |
| UGT1A5 | -0.88 | 1.61E-03 | Unaltered group |
| CYP2C8 | -0.93 | 1.80E-03 | Unaltered group |
| PDGFRA | -0.91 | 1.88E-03 | Unaltered group |
| PCDHAC2 | -0.85 | 2.01E-03 | Unaltered group |
| FAM99A | -1.04 | 2.16E-03 | Unaltered group |
| MOGAT2 | -0.92 | 2.17E-03 | Unaltered group |
| TRHDE | -0.91 | 2.38E-03 | Unaltered group |
| UGT2B15 | -0.89 | 2.44E-03 | Unaltered group |
| UGT1A4 | -1.17 | 2.45E-03 | Unaltered group |
| DEFB1 | -1.07 | 2.54E-03 | Unaltered group |
| CHST9 | -0.91 | 3.34E-03 | Unaltered group |
| CA12 | -0.93 | 3.47E-03 | Unaltered group |
| PRAMEF10 | -1.01 | 3.65E-03 | Unaltered group |
| PADI1 | -0.86 | 3.79E-03 | Unaltered group |
| ETNPPL | -0.85 | 3.91E-03 | Unaltered group |
| ABCB11 | -0.93 | 4.12E-03 | Unaltered group |
| HAL | -0.99 | 4.49E-03 | Unaltered group |
| NRG1 | -0.86 | 4.72E-03 | Unaltered group |
| GFRA1 | -0.88 | 4.98E-03 | Unaltered group |
| AKR1D1 | -0.89 | 5.04E-03 | Unaltered group |
| CYP2B7P | -0.96 | 5.07E-03 | Unaltered group |
| EEF1A2 | -1.23 | 5.16E-03 | Unaltered group |
| UGT1A3 | -0.95 | 5.31E-03 | Unaltered group |
| SERPINA7 | -0.9 | 5.51E-03 | Unaltered group |
| CXCL6 | -1 | 6.35E-03 | Unaltered group |
| ADH1C | -0.93 | 6.51E-03 | Unaltered group |
| CLRN3 | -0.93 | 7.51E-03 | Unaltered group |
| SCUBE1 | -0.91 | 7.64E-03 | Unaltered group |
| GUCY2C | -0.87 | 8.80E-03 | Unaltered group |
| CRP | -1.12 | 9.09E-03 | Unaltered group |
| IP6K3 | -0.87 | 9.69E-03 | Unaltered group |
