# Supplementary material for: The bioinformatics and experimental analysis of AlkB family for prognosis and immune cell infiltration in hepatocellular carcinoma
Source: PeerJ. 2021 Sep 1;9:e12123. doi: 10.7717/peerj.12123 (PMC8418211; doi:10.7717/peerj.12123)
Supplement: Supplemental Information 3 [file peerj-09-12123-s003.docx]

**Table S3.** The cox proportional hazard model of the AlkB family and six immune cells involved in tumor infiltration in HCC.

|  | coef | HR | 95%CI_l | 95%CI_u | p.value |
| --- | --- | --- | --- | --- | --- |
| B_cell | -10.720 | 0.000 | 0.000 | 0.044 | 0.006 |
| CD8_Tcell | -6.773 | 0.001 | 0.000 | 0.142 | 0.006 |
| CD4_Tcell | -6.966 | 0.001 | 0.000 | 1.245 | 0.057 |
| Macrophage | 6.804 | 901.687 | 7.180 | 113236.686 | 0.006 |
| Neutrophil | 3.457 | 31.720 | 0.000 | 3110576.753 | 0.556 |
| Dendritic | 7.142 | 1264.029 | 29.811 | 53596.825 | 0.000 |
| ALKBH1 | 0.388 | 1.474 | 0.844 | 2.574 | 0.173 |
| ALKBH2 | 0.113 | 1.119 | 0.847 | 1.479 | 0.428 |
| ALKBH3 | -0.231 | 0.794 | 0.612 | 1.030 | 0.083 |
| ALKBH4 | *0.462* | 1.587 | 1.007 | 2.502 | 0.046 |
| ALKBH5 | -0.178 | 0.837 | 0.574 | 1.222 | 0.357 |
| ALKBH6 | 0.021 | 1.021 | 0.745 | 1.399 | 0.899 |
| ALKBH7 | -0.198 | 0.821 | 0-600 | 1.122 | 0.215 |
| ALKBH8 | -0.119 | 0.888 | 0.493 | 1.599 | 0.692 |
| FTO | -0.152 | 0.859 | 0.597 | 1.236 | 0.413 |
