# Supplementary figures and images for: The bioinformatics and experimental analysis of AlkB family for prognosis and immune cell infiltration in hepatocellular carcinoma
Source: PeerJ. 2021 Sep 1;9:e12123. doi: 10.7717/peerj.12123 (PMC8418211; doi:10.7717/peerj.12123)

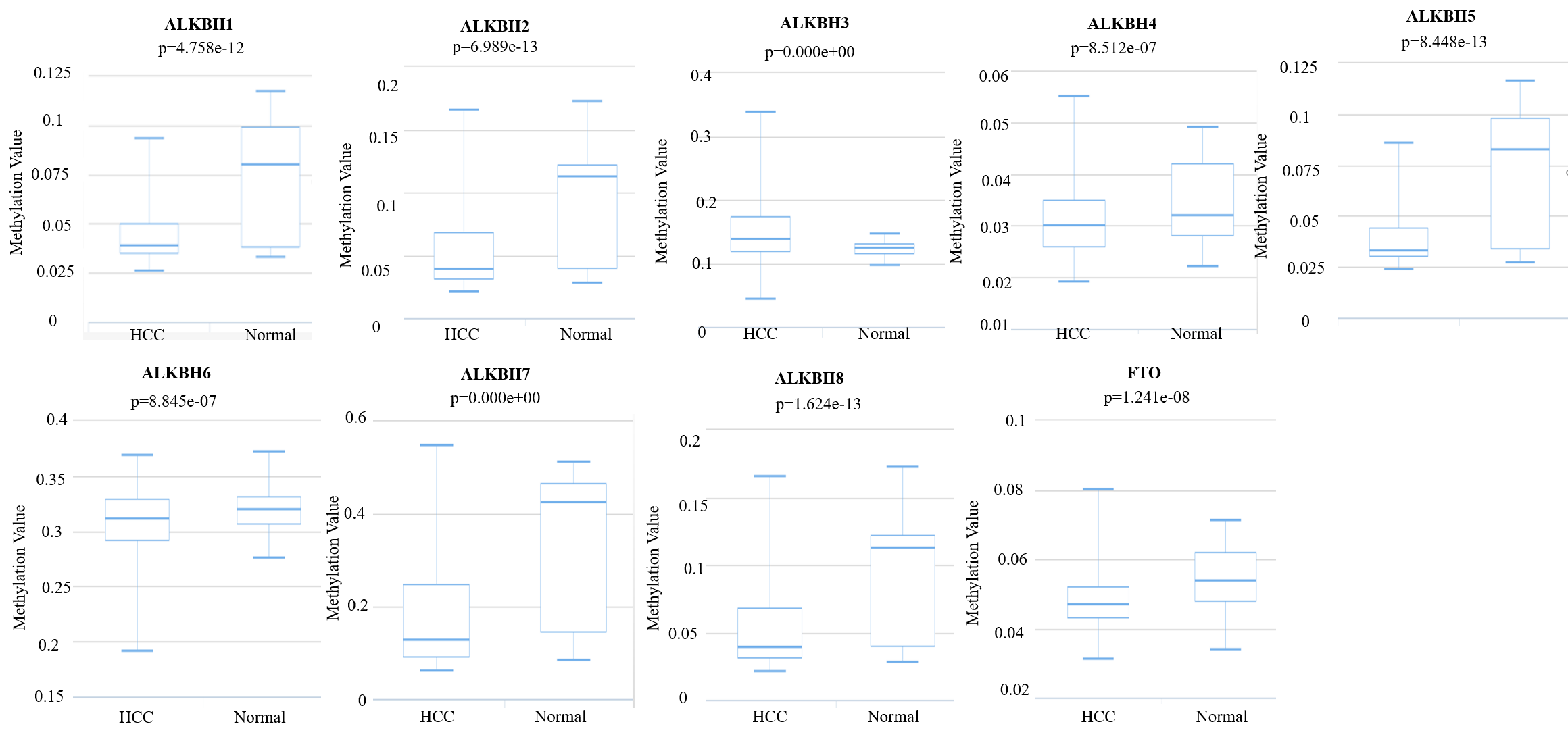

Supplement: Supplemental Information 4 [file peerj-09-12123-s004.png]
